# Difference in oxidative stress tolerance between rice cultivars estimated with chlorophyll fluorescence analysis

**DOI:** 10.1186/s13104-017-2489-9

**Published:** 2017-04-26

**Authors:** Ichiro Kasajima

**Affiliations:** 1 Institute of Molecular and Cellular Biosciences, University of Tokyo, Yayoi 1-1-1, Bunkyo-ku, Tokyo, Japan; 2 Department of Agriculture, Iwate University, Ueda 3-18-8, Morioka, Iwate Japan

**Keywords:** Chlorophyll fluorescence, Co13, Koshihikari, Methyl viologen, Modern cultivar, Oxidative stress, Rice

## Abstract

**Background:**

Oxidative stress is considered to be involved in growth retardation of plants when they are exposed to a variety of biotic and abiotic stresses. Despite its potential importance in improving crop production, comparative studies on oxidative stress tolerance between rice (*Oryza sativa* L.) cultivars are limited. This work describes the difference in term of oxidative stress tolerance between 72 rice cultivars.

**Methods:**

72 rice cultivars grown under naturally lit greenhouse were used in this study. Excised leaf discs were subjected to a low concentration of methyl viologen (paraquat), a chemical reagent known to generate reactive oxygen species in chloroplast. Chlorophyll fluorescence analysis using a two-dimensional fluorescence meter, ion leakage analysis as well as the measurement of chlorophyll contents were used to evaluate the oxidative stress tolerance of leaf discs. Furthermore, fluorescence intensities were finely analyzed based on new fluorescence theories that we have optimized.

**Results:**

Treatment of leaf discs with methyl viologen caused differential decrease of maximum quantum yield of photosystem II (*F*v/*F*m) between cultivars. Decrease of *F*v/*F*m was also closely correlated with increase of ion leakage and decrease of chlorophyll *a*/*b* ratio. *F*v/*F*m was factorized into photochemical and non-photochemical parameters to classify rice cultivars into sensitive and tolerant ones. Among the 72 compared rice cultivars, the traditional cultivar Co13 was identified as the most tolerant to oxidative stress. Koshihikari, a dominant modern *Japonica* cultivar in Japan as well as IR58, one of the modern *Indica* breeding lines exhibited a strong tolerance to oxidative stress.

**Conclusions:**

Close correlation between *F*v/*F*m and chlorophyll *a*/*b* ratio provides a simple method to estimate oxidative stress tolerance, without measurement of chlorophyll fluorescence with special equipment. The fact that modern cultivars, especially major cultivars possessed tolerance to oxidative stress suggests that oxidative stress tolerance is one of the agricultural traits prerequisite for improvement of modern rice cultivars. Data presented in this study would enable breeding of rice cultivars having strong tolerance to oxidative stress.

**Electronic supplementary material:**

The online version of this article (doi:10.1186/s13104-017-2489-9) contains supplementary material, which is available to authorized users.

## Background

Ancient Asian people selected mutations in rice genome which cause desirable features either to the whole rice plant architecture or to its grain. Selection during rice domestication helped to identify mutations affecting traits such as grain shattering, grain pericarp color, sticky grains, grain size/shape, grain fragrance, grain number, and semi-dwarfism [1, 2]. However, it is not clear whether other physiological traits were selected during rice domestication. We previously reported a physiological difference between rice subclasses, we showed, a higher capacity of non-photochemical quenching in *Japonica* cultivars compared with *Indica* cultivars [3]. Although the direct impact on agriculture of such increase of non-photochemical quenching in *Japonica* cultivars is not yet clear, we speculate its involvement in the process of acclimation to cool climates. A mutation responsible for non-photochemical quenching would have contributed to give rise to *Japonica* cultivars, rather than having been selected throughout cultivation.

Modern cultivars are bred by crossing between traditional cultivars and selection of progenies having superior agricultural traits than traditional cultivars. Modern high-yield cultivars are also highly responsive to fertilizer application [4]. Then, the question is whether other physiological traits that characterize quality of modern rice cultivars can be identified. One of such potential agricultural trait may be anti-oxidative capacity. Reactive oxygen species are generated under various stresses in rice plant [5–8]. Among them, illumination of sunlight catalyzes generation of reactive oxygen species in plant (called photo-oxidation) [9, 10]. Improved oxidative stress tolerance of rice plant is expected to benefit fitness of rice plant either under harsh or mild climates. General knowledge on difference (or similarity) in oxidative stress tolerance between cultivars is the first step toward understanding its significance in rice breeding. In fact, environmental stress tolerance emerges as one of the factors enabling fertilizer response of high-yield cultivars [4].

Because oxidative stress rises as a secondary stress, induced by other stresses such as heat, cold, salt, drought, aluminum and high light [5–9, 11], it is difficult to clearly evaluate the effect of oxidative stress, and the importance of anti-oxidative capacity of crops, despite its potential importance in agriculture. In this study methyl viologen (also known as paraquat) was adopted as an artificial inducer of oxidative stress in rice leaves, like many previous reports. Methyl viologen is one of the most frequently used inducer of oxidative stress in plant studies. Here we report the difference of tolerance to oxidative stress between 72 rice cultivars following the treatment of leaf discs with methyl viologen.

## Methods

### Plant materials

Seeds of ‘World Rice Core Collection’ were obtained from National Institute of Agrobiological Sciences, Japan. This Collection reflects the genetic diversity of all rice cultivars [12]. Classification of rice cultivars into four subgroups was done based on previous report [3, 12]. Seeds were incubated in growth chamber at 28 °C for 4–5 days then transferred to pots containing nutrient-rich soil (Bon-sol #1, Sumitomo Chemical, Tokyo, Japan). Plants were grown in a naturally lit greenhouse in the building of the Institute of Molecular and Cellular Biosciences in 2008 and 2009. Temperature in greenhouse was 28 °C in the day (16 h) and 24 °C in the night (8 h). The light intensity (photosynthetic photon flux density, PPFD) in the greenhouse was variable according to climates, with the maximum intensity of around 1000 μmol-photons m^−2^ s^−1^. Although, about half of sunlight is absorbed by glass in the greenhouse, this light intensity (1000 μmol-photons m^−2^ s^−1^) is still much higher than intensity in growth chambers, the reason why rice seedlings are commonly pre-germinated in greenhouse by Japanese farmers. All cultivars were allowed to grow under the same spacial orientation in the absence of any shade to ensure uniform growth, to avoid potential differential light intensity effects on cultivars throughout the experiments. Some cultivars, which showed poor growth under these conditions were not considered in this study. Data were reproducible independently from the plant growth stage. Cultivars Koshihikari and Co13 shown in Fig. 3e, were pre-germinated in water for 6 days followed by a growth in half-strength Murashige-Skoog culture salt containing 0, 0.01, 0.1 or 1 μM methyl viologen for 4 days.

### Chlorophyll fluorescence

**Fig. 1:**
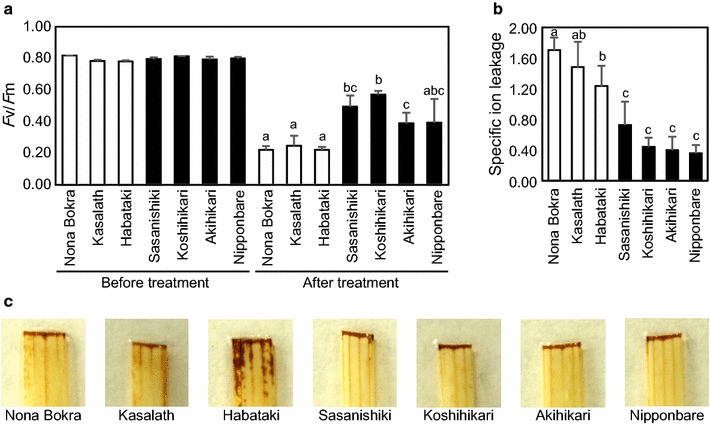
Oxidative stress tolerance in representative seven rice cultivars. **a** *F*v/*F*m values before and after treatment with methyl viologen. **b** Specific ion leakage induced by treatment with methyl viologen. In **a** and **b**, *Indica* cultivars are shown with *white bars*, and *Japonica* cultivars are shown with *black bars*. Data represent means and standard deviations. *n* = 4. Student’s *t* test was performed on ‘after treatment’ data in (**a**) and data in (**b**). *Different alphabets up to bars* indicate significant difference between cultivars (P < 0.05). **c** Leaves stained with 3,3′-diaminobenzidine

**Fig. 2:**
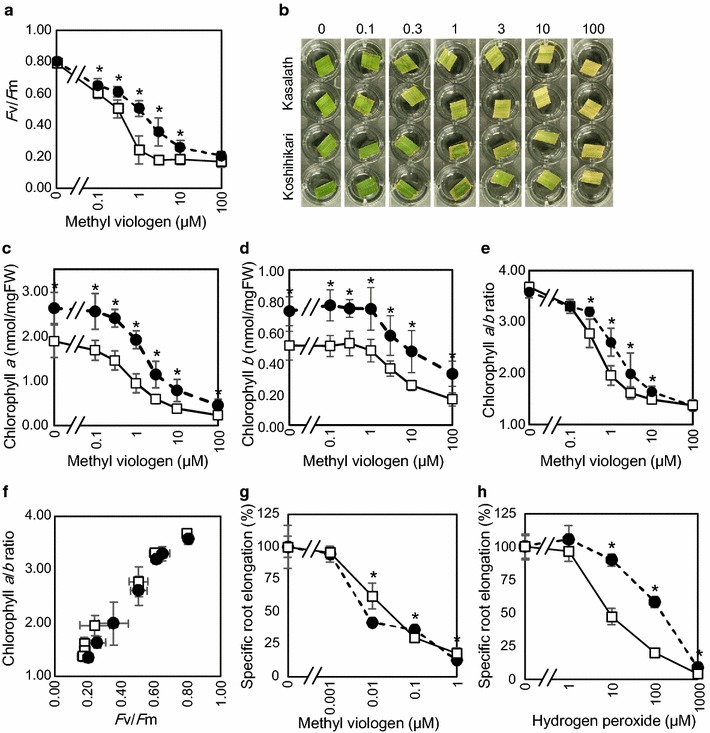
Comparison between tolerant and sensitive cultivars with various methods. **a** *F*v/*F*m values after treatment with a series of concentrations of methyl viologen. **b** Photographs of a part of leaf discs treated with methyl viologen for 37 h. *Numbers up to each photograph* indicate concentrations of methyl viologen (μM). **c** Chlorophyll *a* content, **d** chlorophyll *b* content, and **e** chlorophyll *a*/*b* ratio of leaf discs after treatment with methyl viologen. **f** Relationship between *F*v/*F*m measured in **a** and chlorophyll *a*/*b* ratio measured in **e**. In **a** through **f**, *white square* indicates Kasalath (sensitive cultivar) and *black circle* indicates Koshihikari (tolerant cultivar). Data represent means and standard deviations. *n* = 8. **g** Specific root elongation in solutions containing methyl viologen. **h** Specific root elongation in solutions containing hydrogen peroxide. In **g** and **h**, *white square* indicates Kasalath (sensitive cultivar), and *black circle* represents Nipponbare (moderately tolerant cultivar). Data represent means and standard deviations. *n* = 5. In **a**, **c**, **d**, **e**, **g** and **h**, *asterisks* indicate statistical difference between cultivars judged by Student’s *t* test (P < 0.05)

**Fig. 3:**
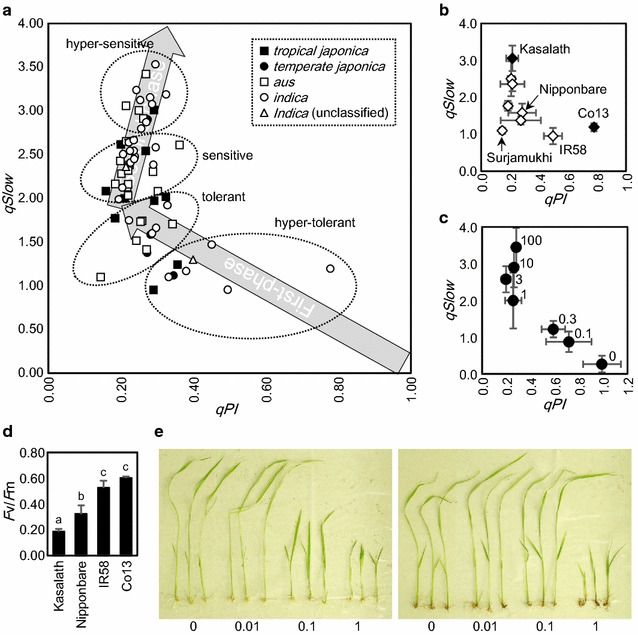
Estimation of oxidative stress tolerance of World Rice Core Collection. **a** *qPI* and *qSlow* values of 67 cultivars. Data represent means of four replications. **b** The same data as **a**, of selected cultivars. *White diamonds* indicate all seven modern cultivars analyzed in this experiment. Data represent means and standard deviations. **c** *qPI* and *qSlow* values of Nipponbare treated with a series of concentrations of methyl viologen. Concentrations of methyl viologen are shown to the *right of each plot*. Data represent means and standard deviations. *n* = 4. **d** *F*v/*F*m values of four cultivars, after treatment with methyl viologen. Data represent means and standard deviations. *n* = 4. *Different alphabets up to bars* indicate statistical difference by Student’s *t* test (P < 0.05). **e** Koshihikari (*left*) and Co13 (*right*) seedlings grown in media containing methyl viologen. Concentrations of methyl viologen in media are indicated under photographs (μM)

About 6 mm leaf discs were excised from the center of fully expended leaf blades of independent rice plants at 8, 12 and 4 weeks after germination (Figs. 1, 2, 3 respectively), and immediately dipped into 2-mL solutions using 24-well plastic plates. Solutions of Triton X-100 at 0.01% and methyl viologen at different concentrations (0.1; 0.3;1;3;10 and 100 μM) were used. The concentration of methyl viologen reported here (1 μM) is ten times lower than previously report [13]. Preliminary experiments allowed the determination of this optimum concentration of 1 μM under which we could detect difference between cultivars. Duration of illumination with low-intensity light was extended from 6 h to 20 h in place. *F*v/*F*m value which quantify chlorophyll fluorescence was around 0.8 in all tested cultivars before start of illumination. This value, of 0.8, was also observed in untreated leaves throughout all experiments. Values around 0.8 were also observed for control leaves treated with water (without methyl viologen). The only exception was recorded in cv. Surjamukhi, which is plotted at the most-left side of in Fig. 3a and b. Leaves of cv. Surjamukhi were in fact damaged even when treated with water. Leaves were dark-adapted for 5 min before measuring *F*v/*F*m value using a two-dimensional fluorescence imager (Closed FluoroCam, Photon Systems Instruments, Brno, Czech Republic). After light treatment using white LED light (PPFD = 120 μmol-photons m^−2^ s^−1^ in Fig. 1a and PPFD = 150 μmol-photons m^−2^ s^−1^ in the other figures), *F*v/*F*m was measured again, after being dark-adapted for 5 min. *F*v/*F*m values measured after light treatment (photo-damage catalyzed by methyl viologen) were smaller than those before the start of light treatment. Duration of light treatment was 18 h in the analysis of World Rice Core Collection, and 20 h in the other analyses. Calculations of *F*v/*F*m, *qPI*, *qSlow* and fluctuation of the sensitivity factor were done as described in our previous reports [14, 15]. Calculation of *qPI* and *qSlow* required further measurements before and after photo-damage.

### Chlorophyll contents

Leaf discs were soaked in *N*,*N*-dimethylformamide overnight under dark conditions at 10 °C. Chlorophylls *a* and *b* were photometrically quantified [16].

### Ion leakage

Leaf discs excised from expanded leaves of independent mature plants (16 w after germination) were placed on 2-mL solutions containing 0.01% Triton X-100 and 1 μM methyl viologen. After 30 min vacuum, leaf discs were put under light at 150 μmol-photons m^−2^ s^−1^ for 16 h. ‘Specific ion leakage’ was calculated from electronic conductivity of solution before (EC_b_) and after illumination (EC_a_) as follows:

$$\left( {\text{Specific ion leakage}} \right) = \left( {{\text{EC}}_{\text{a}} {-}{\text{EC}}_{\text{b}} } \right)/{\text{EC}}_{\text{b}}$$

### Staining

Hydrogen peroxide accumulated in leaves was stained as follows. Largest leaf blades of juvenile plants (2 w after germination) were excised near the center, and floated on solution containing 1 mg mL^−1^ 3,3′-diaminobenzidine. pH of the solution was adjusted to 3.8. After light treatment at medium intensity (PPFD = 600 μmol-photons m^−2^ s^−1^) for 7 h, leaves were boiled in ethanol to remove pigment. 3,3′-diaminobenzidine is precipitated as insoluble dark-brown polymer when oxidized by hydrogen peroxide.

### Root elongation

Independent rice seeds used to quantify root elongation (in Fig. 2g, h) were germinated for 3 days in water. Germinated rice plants were transferred to plastic mesh floating on solution containing 0.5 mM CaCl_2_, 1 mM potassium phosphate buffer (pH = 5.8), and methyl viologen (ranging from 0.001 to 1 μM) or hydrogen peroxide (ranging from 1 to 1000 μM). ‘Specific root elongation’ was calculated by dividing each value by the average elongation in the absence of stress. Specific root elongation of 3 days-germinated plants was observed for 24 h for cv. Kasalath and 47 h for cv. Nipponbare.

## Results

### Oxidative stress tolerance of seven rice cultivars

In this report, tolerance of rice cultivars to oxidative stress was compared. Seven rice cultivars extensively studied in Japan including three *Indica* cultivars (Nana Bokra, Kasalath and Habataki) and four *Japonica* cultivars (Sasanishiki, Koshihikari, Akihikari and Nipponbare) were tested. Potential difference in oxidative stress tolerance was first investigated. Leaf discs were floated on water containing detergent and methyl viologen. This mild treatment was appropriate to observe differential responses between cultivars, which was reflected by the reduction in *F*v/*F*m values (Fig. 1a). This reduction in *F*v/*F*m value was significantly larger in the three *Indica* cultivars than in the four *Japonica* cultivars. Moreover, oxidative stress tolerance of the seven cultivars showed the same tendency based on their specific ion leakage (Fig. 1b). Data on the accumulation of hydrogen peroxide, show that the leaves of sensitive cultivars to oxidative stress tend to accumulate more hydrogen peroxide (Fig. 1c).

### Comparison between sensitive and tolerant cultivars at various concentrations of methyl viologen

Next, a tolerant cultivar Koshihikari and a sensitive one Kasalath were compared. Leaf discs excised from plants were put under light conditions for 20 h on water containing a series of concentrations of methyl viologen. Difference in *F*v/*F*m value was the largest at 1 μM (Fig. 2a). Treatment of these leaves were extended to 37 h to allow chlorophyll degradation, time after which leaves treated with high concentration of methyl viologen turned from green to yellow (Fig. 2b). Both chlorophyll *a* and *b* were degraded by oxidative stress (Fig. 2c, d). Interestingly, chlorophyll *b* was not degraded when intermediate concentrations (0.3 and 1 μM) of methyl viologen were used although chlorophyll *a* was degraded under these same concentrations. This imbalanced degradation of chlorophylls resulted in a serial decrease of chlorophyll *a*/*b* ratio, depending on both the concentrations of methyl viologen and on rice cultivar (Fig. 2e). A comparison between *F*v/*F*m values (after 20 h of treatment) and chlorophyll *a*/*b* ratio (37 h treatment), showed a clear correlation between them (Fig. 2f).

The response of roots to chemicals treatment was also assessed using a moderately tolerant cultivar Nipponbare and a sensitive cultivar Kasalath. According to Fig. 2g, a relatively low concentrations of Methyl viologen (such as 0.01 μM) significantly inhibited root elongation, and this was to a similar extent for both Kasalath and Nipponbare. Root elongation was also measured under the presence of hydrogen peroxide in culture. Nipponbare showed significantly higher tolerance to hydrogen peroxide than Kasalath (Fig. 2h).

### Oxidative stress tolerance of World Rice Core Collection

Sixty-seven cultivars of World Rice Core Collection, including Kasalath and Nipponbare, were analyzed by chlorophyll fluorescence. Treatment with Methyl viologen caused *F*v/*F*m values of many cultivars to decrease down to nearly 0.2 (Table 1). In order to compare tolerance of such sensitive cultivars, *F*v/*F*m was factorized into two parameters *qPI* and *qSlow*. *qPI* value represents changes in photochemical de-excitation processes, and *qSlow* represents changes in non-photochemical de-excitation processes [14]. *qPI*–*qSlow* plot for all cultivars is shown in Fig. 3a. Standard deviations are not shown to simplify the figure. Four subgroups (*Japonica* subgroups, including *tropical japonica* and *temperate japonica*; and *Indica* subgroups including *aus* and *indica*) are indicated with different symbols. We observed harmonized changes in *qPI* and *qSlow* values in the course of photo-damage of rice leaves with our previous report [15]. A similar decrease in *qPI* value accompanied by increase in *qSlow* value was also observed in response to methyl viologen in the present analysis.

**Table 1:** Chlorophyll fluorescence parameters of World Rice Core Collection

Cultivar	Classification	Tolerance	*qPI*		*qSlow*		*F*v/*F*m	
			Average	SD	Average	SD	Average	SD
Nipponbare	*temperate j.*	Tolerant	0.28	0.10	1.59	0.25	0.32	0.07
Kasalath	*aus*	Hyper-sensitive	0.21	0.04	3.06	0.34	0.19	0.02
Bei Khe	*indica*	Hyper-sensitive	0.25	0.02	3.12	0.19	0.21	0.02
Jena 035	*aus*	Tolerant	0.24	0.08	1.51	0.36	0.28	0.09
Naba	*indica*	Tolerant	0.30	0.04	1.66	0.36	0.32	0.06
Puluik Arang	*indica*	Hyper-sensitive	0.26	0.06	2.80	0.57	0.21	0.03
Davao 1	*indica*	Hyper-sensitive	0.27	0.05	2.87	0.30	0.23	0.05
Ryou Suisan Koumai	*indica*	Hyper-tolerant	0.45	0.10	1.47	0.27	0.44	0.09
Shuusoushu	*indica*	Hyper-sensitive	0.28	0.05	3.15	0.66	0.23	0.01
Jinguoyin	*indica*	Tolerant	0.33	0.03	1.92	0.69	0.33	0.07
Dahonggu	Indica**	Hyper-tolerant	0.40	0.12	1.30	0.78	0.44	0.14
IR 58	*indica*	Hyper-tolerant	0.50	0.06	0.95	0.22	0.53	0.05
Co 13	*indica*	Hyper-tolerant	0.78	0.01	1.19	0.11	0.60	0.01
Vary Futsi	*indica*	Hyper-sensitive	0.29	0.06	3.08	0.92	0.23	0.02
Keiboba	*indica*	Hyper-sensitive	0.24	0.02	3.24	0.32	0.20	0.03
Qingyu	*indica*	Sensitive	0.23	0.05	2.41	0.36	0.21	0.04
Deng Pao Zhai	*indica*	Sensitive	0.21	0.02	2.51	0.47	0.20	0.03
Tadukan	*indica*	Sensitive	0.22	0.03	2.64	0.17	0.20	0.02
Shwe Nang Gyi	*indica*	Hyper-sensitive	0.30	0.04	3.53	0.25	0.21	0.02
Calotoc	*aus*	Sensitive	0.22	0.04	2.65	0.44	0.20	0.02
Lebed	*indica*	Hyper-sensitive	0.32	0.02	3.19	0.57	0.21	0.02
Pinulupot 1	*indica*	Sensitive	0.23	0.03	2.54	0.27	0.21	0.02
Muha	*aus*	Tolerant	0.34	0.09	1.70	0.28	0.35	0.08
Jhona 2	*aus*	Sensitive	0.22	0.05	2.13	0.85	0.21	0.01
Nepal 8	*aus*	Sensitive	0.30	0.12	2.08	0.40	0.27	0.09
Jarjan	*aus*	Sensitive	0.36	0.09	2.61	1.66	0.33	0.17
Kalo Dhan	*aus*	Hyper-sensitive	0.25	0.03	3.00	0.55	0.21	0.02
Anjana Dhan	*aus*	Sensitive	0.29	0.02	2.30	0.48	0.27	0.03
Shoni	*aus*	Sensitive	0.20	0.04	2.28	0.49	0.21	0.02
Tupa 121-3	*aus*	Sensitive	0.22	0.03	2.38	0.28	0.23	0.02
Surjamukhi	*aus*	Tolerant	0.14	0.00	1.10	0.08	0.23	0.00
ARC 7291	*aus*	Hyper-sensitive	0.26	0.02	2.91	0.31	0.23	0.03
ARC 5955	*aus*	Sensitive	0.22	0.04	2.32	0.26	0.21	0.02
Ratul	*aus*	Sensitive	0.22	0.04	2.03	0.65	0.24	0.02
ARC 7047	*aus*	Tolerant	0.27	0.09	1.41	0.41	0.32	0.11
ARC 11094	*aus*	Hyper-sensitive	0.27	0.03	3.42	0.33	0.20	0.02
Badari Dhan	*aus*	Sensitive	0.19	0.04	2.16	0.51	0.19	0.01
Nepal 555	*aus*	Tolerant	0.26	0.08	1.74	0.83	0.27	0.10
Kaluheenati	*aus*	Sensitive	0.22	0.02	2.16	0.50	0.21	0.02
Local Basmati	*aus*	Sensitive	0.20	0.03	2.43	0.39	0.20	0.03
Dianyu 1	*temperate j.*	Tolerant	0.27	0.14	1.38	0.11	0.32	0.12
Basilanon	*aus*	Tolerant	0.25	0.03	1.73	0.72	0.27	0.03
Ma sho	*tropical j.*	Sensitive	0.16	0.02	2.08	0.20	0.17	0.02
Khao Nok	*tropical j.*	Hyper-sensitive	0.29	0.04	3.01	0.27	0.23	0.03
Jaguary	*tropical j.*	Hyper-tolerant	0.29	0.12	0.95	0.63	0.37	0.17
Khau Mac Kho	*tropical j.*	Sensitive	0.20	0.07	2.62	0.44	0.19	0.04
Padi Perak	*tropical j.*	Tolerant	0.32	0.25	2.02	0.65	0.29	0.19
Rexmont	*tropical j.*	Tolerant	0.18	0.03	1.77	0.13	0.22	0.02
Urasan 1	*tropical j.*	Tolerant	0.29	0.04	1.97	0.20	0.28	0.03
Khau Tan Chiem	*tropical j.*	Sensitive	0.27	0.10	2.54	0.39	0.24	0.05
Tima	*tropical j.*	Hyper-tolerant	0.36	0.19	1.24	0.42	0.37	0.15
Tupa729	*tropical j.*	Sensitive	0.21	0.04	2.00	0.34	0.22	0.02
Milyang 23	Indica**	Sensitive	0.21	0.08	2.36	0.19	0.20	0.06
Neang Menh	*indica*	Sensitive	0.30	0.04	2.58	0.50	0.23	0.01
Neang Phtong	*indica*	Sensitive	0.29	0.04	2.39	0.22	0.25	0.04
Hakphaynhay	*indica*	Hyper-tolerant	0.38	0.11	1.17	0.21	0.40	0.08
Radin Goi Sesat	*indica*	Sensitive	0.24	0.07	2.67	0.84	0.20	0.02
Kemasin	*indica*	Sensitive	0.24	0.05	2.48	0.43	0.22	0.05
Bleiyo	*indica*	Sensitive	0.20	0.05	2.12	0.76	0.21	0.04
Padi Kuning	*indica*	Sensitive	0.24	0.03	2.54	0.41	0.21	0.02
Rambhog	*indica*	Tolerant	0.22	0.05	1.75	0.73	0.25	0.06
Bingala	*indica*	Tolerant	0.29	0.09	1.60	0.68	0.32	0.13
Phulba	*temperate j.*	Hyper-sensitive	0.27	0.07	2.90	0.59	0.23	0.03
Khao Nam Jen	*temperate j.*	Hyper-tolerant	0.35	0.10	1.12	0.51	0.38	0.11
Chin Galay	*indica*	Sensitive	0.24	0.10	2.45	0.50	0.22	0.06
Hong Cheuh Zai	*indica*	Hyper-tolerant	0.33	0.07	1.10	0.56	0.41	0.10
Vandaran	*indica*	Sensitive	0.19	0.03	1.99	0.11	0.21	0.03

*n* = 4

*SD* standard deviation

** Unclassified *Indica* cultivars

Trace of *qPI*–*qSlow* coordinates during photo-damage is as follows: Because healthy leaves have 100% capacity of photochemistry (*qPI*) and no induction of slow-relaxing non-photochemical quenching (*qSlow*), coordinate (1, 0) was defined as the initial coordinate. Upon treatment, coordinates rise semi-linearly to the left, to reach (0.2, 2). Then for some reason that will be discussed later, coordinates change direction to the right, to direct to (0.4, 5). This bi-phasic changes in *qPI* and *qSlow* values is illustrated with light-gray arrows in Fig. 3a. Notably, cultivars plotted near the original point (1, 0) are tolerant and those plotted near arrow-head are sensitive. Cultivars were classified as hyper-tolerant (9 cultivars), tolerant (15 cultivars), sensitive (29 cultivars) or hyper-sensitive (14 cultivars) according to their positions along the arrows.

*qPI*–*qSlow* plot of some selected cultivars are shown in Fig. 3b. A traditional cultivar Co13 came from India was the most tolerant to oxidative stress, followed by a modern cultivar IR58 from Philippines. Four other modern cultivars (Rexmont from U.S.A., Nipponbare from Japan, Dianyu1 from China and Surjamukhi from India) were tolerant, and the other two modern cultivars (Deng Pao Zhai from China and Milyang23 from South Korea) were sensitive. It’s noteworthy that no modern cultivar examined in this study was hyper-sensitive to oxidative stress. Also, ratio of tolerant cultivars is higher in modern cultivars (1 hyper-tolerant, 4 tolerant and 2 sensitive) compared with traditional cultivars (6 hyper-tolerant, 9 tolerant, 27 sensitive and 14 hyper-sensitive cultivars).

Changes in *qPI* and *qSlow* values were also observed in Nipponbare treated with different concentrations of methyl viologen (Fig. 3c). Leaf discs were plotted on the same bi-phasic line as shown in Fig. 3a, confirming previous observations as well as the reproducibility of this change. *F*v/*F*m values of Kasalath, Nipponbare, IR58 and Co13 after treatment with methyl viologen are shown in Fig. 3d. These values ranged from 0.2 to 0.6. In Fig. 3e, Koshihikari and Co13 were grown in nutrient solution containing methyl viologen.

## Discussion

The oxidative stress tolerance of 72 rice cultivars treated with 1 μM of methyl viologen was reported in this study. Methyl viologen is one of the commonly used reagent for the induction of oxidative stress in plant. This relatively low concentration of methyl viologen was enough to cause damage to photosynthetic machinery, which could be observed by the responses of chlorophyll fluorescence parameters. The use of similar or lower concentration of methyl viologen has previously been reported. This study shows that the effect of 1 μM of methyl viologen was more toxic to root growth. This toxicity neither seems to be linked to photo-damage nor to be useful to detect difference between cultivars. Such secondary effect of methyl viologen on leaves could have been minimized. On the other hand, hydrogen peroxide seems to be useful for comparison of oxidative stress tolerance of rice root. Direct treatment of leaf discs with methyl viologen allowed a better estimation of oxidative stress tolerance in leaf tissues, compared with the indirect application of methyl viologen to culture medium, where difference in rates of absorption and translocation of methyl viologen would have affected tolerance of rice plants. Although studies on differential uptake and translocation of methyl viologen in plant are lacking, a clear image can emerge based on the correlation between uptake and toxicity of other toxic compounds such as selenate and germanium [17, 18].

*F*v/*F*m value provides an appropriate estimation of damage caused by methyl viologen. This was demonstrated through the reasonable correlation between values of *F*v/*F*m and ion leakage (Fig. 1a, b), and between values of *F*v/*F*m and chlorophyll *a*/*b* ratio (Fig. 2f). Furthermore, the correlation between *F*v/*F*m and chlorophyll *a*/*b* ratio suggests that tolerant cultivars to oxidative stress, estimated by *F*v/*F*m values in this study, would be able to protect chlorophyll pigment from breakdown by oxidative stress. Moreover, sensitive lines to oxidative stress tended to accumulate more hydrogen peroxide in intact leaves compared with tolerant ones (Fig. 1c). The decrease in chlorophyll *a*/*b* ratio under photo-damage is useful for future studies. Measurement of this ratio is simple and thus could be a good indicator to evaluate damage caused by oxidative stress, especially when special tool for fluorescence measurement based on ‘pulse amplitude modulation’ technique is not available in the laboratory or in the field. Favorable degradation of chlorophyll *a* may happen because chlorophyll *a* is bound to photosystems I and II, where light reaction takes place, whereas chlorophyll *b* is bound to antenna complex. Decrease in chlorophyll *a*/*b* ratio is also observed in shaded leaves. This is an adaptive mechanism to absorb broad spectra of light [19]. Chlorophyll *a*/*b* ratio may also have effect on leaf colors. Although the exact colors of chlorophyll *a* and chlorophyll *b* are not clear, chlorophyll *a* would be bluish green and chlorophyll *b* would be yellowish green, judging from their absorbance spectra and RGB color matching function [20, 21]. Decrease in chlorophyll *a*/*b* ratio will be one of the reasons for leaf yellowing under oxidative stress, together with dichromatism caused by decreased chlorophyll concentrations [21], and yellow xanthophyll pigments [20]. Further information about the exact color of each of the chlorophylls *a* and *b* are needed for better understanding of this phenomenon.

Technical advantage of using *F*v/*F*m as mean of estimation of photo-damage is the speed of its analysis and its applicability to small samples. For measurement of ion leakage, leaf discs had to be excised from mature plants because there is no enough amount of ion in young leaves (according to our preliminary experiments). Furthermore, it offers the advantage of measuring 24 samples at once, using two-dimensional fluorescence imager. Similar decrease of *F*v/*F*m was observed under the same treatments with methyl viologen, regardless of growth stages of rice plants (juvenile, young or mature). These advantages of the method allowed simultaneous and reliable estimation of anti-oxidative capacity of ‘World Rice Core Collection’.

The bi-phasic and correlated change of *qPI* and *qSlow* values in Fig. 3 is consistent with previous finding. For instance, a similar change of coordinate from (1, 0) to (0.2, 2) caused by photo-damage was observed in our previous report [15]. In addition to this first-phase move of plot, a second-phase move of plot from (0.2, 2) to (0.4, 5) was found in this study. *F*v/*F*m does not change in this second phase. In other words, *F*v/*F*m value is negatively correlated with the slope of line connecting between (0, −1) and plot of leaves. The second phase of move occurs on one of such lines, connecting between (0, −1) and (0.2, 2). Simultaneous and proportional increase in photochemical process (*qPI*) and non-photochemical process (1 + *qSlow*) occurs when the value of the sensitivity factor fluctuates [15]. Fluctuation of the sensitivity factor would have occurred because of decrease in light absorption of leaf, as a result of chlorophyll breakdown. When *qSlow* value changes from 2 to 3.5, proportion of this fluctuation (*σ*) is:

$$\sigma = \left( { 1+ qSlow} \right)/\left( { 1+ qSlow^{\prime }} \right) = \left( { 1+ 2} \right)/\left( { 1+ 3. 5} \right) = 2/ 3$$

Thus, *qPI* and *qSlow* values proportionally increased on a second-phase regression line, in rice leaves with only low anti-oxidative capacity. In this course, light absorption decreased down to approximately 67% in cultivars with the lowest anti-oxidative capacities, namely Shwe Nang Gyi and ARC11094. Apart from chlorophyll breakdown, it is not yet clear why *qSlow*, the slow-relaxing non-photochemical quenching is induced accompanied by photo-damage. Chloroplast movement could explain part of it [22], but its contribution is not high when fluorescence is measured in two dimension [15]. It may be worth considering the effect of imbalanced proportion of chlorophyll *a*/*b* ratio for induction of *qSlow*, which was observed in this report.

*qPI* and *qSlow* values enabled classification of rice cultivars into four classes (hyper-tolerant, tolerant, sensitive and hyper-sensitive). Estimation of anti-oxidative capacity of similar set of rice cultivars, by treatment of single plants per cultivar with high concentration of methyl viologen added to media was already reported [23]. Despite the large differences in experimental conditions, these two reports have some trend in common. In this study, *F*v/*F*m values after treatment with methyl viologen are typically 0.4–0.6 for hyper-tolerant cultivars, 0.3 for tolerant cultivars, and 0.2 for sensitive/hyper-sensitive cultivars (Table 1). In this sense, Koshihikari is also hyper-tolerant to oxidative stress. Co13 was more tolerant to oxidative stress than Koshihikari (Fig. 3e). By contrast to capacity of non-photochemical quenching, there was no discrimination of anti-oxidative capacity between rice subclasses or subgroups (Fig. 3a). Different kinds of genes are needed to maintain level of oxidative stress tolerance (such as ascorbate peroxidase, glutathione peroxidase, glutathione reductase, heme oxygenase, Ndh complex, lipocalin, basic helix-loop-helix transcription factor and PsbS) [24–33]. Similar set of genes also improve plant tolerance to oxidative stress when ectopically expressed by transformation (superoxide dismutase, ascorbate peroxidase, monodehydroascorbate reductase, catalase, glutathione peroxidase, aldehyde dehydrogenase, Nudix hydrolase, thioredoxin, nucleoside diphosphate kinase, 2-cysteine peroxiredoxin, methionine sulfoxide reductase, Fe-chelatase, bZIP transcription factor, and annexin) [34–48]. Some of these genes improve cold, heat, salt or heavy metal tolerance as well, further supporting importance of oxidative stress tolerance in adaptation to abiotic stresses. Localization of enzymes in chloroplast is also key to oxidative stress tolerance for some of the genes (superoxide dismutase, ascorbate peroxidase, glutathione-*S*-transferase, glutathione reductase, dehydroascorbate reductase, ferredoxin-NADP(H) reductase, Nudix hydrolase, RecA, and flavodoxin) [49–56]. Many other genes (including ferritin and miR398) are also regulated by oxidative stress [57–62]. However, contribution to oxidative stress tolerance is not only restricted to genes, and metabolites (proline, salicylic acid, ascorbic acid, glutathione, and vitamin B_6_) also improve oxidative stress tolerance [63–70]. Considering such large number of genes and metabolites contributing to oxidative stress tolerance, it is not strange that there was no discrimination in oxidative stress tolerance between *Indica* and *Japonica*. Rice cultivars may have evolved their own tolerance mechanisms by accumulating mutations in these genes. Roughly speaking, there also seems to be a ‘trade-off’ between growth rate and oxidative stress tolerance. Cultivars such as Kasalath and Nona Bokra grow much faster than Japanese cultivars (for instance Koshihikari), whereas Nona Bokra accumulates lower concentration of metabolites than Koshihikari in leaf (Kasajima, unpublished data). This will be also correlated with lower chlorophyll contents in Kasalath (Fig. 2). However, this is just a hypothesis, and mechanism of oxidative stress tolerance should be examined from various aspects.

Unlike traditional cultivars mainly consisting of sensitive cultivars, many of the modern cultivars were tolerant to oxidative stress. Koshihikari is a dominant cultivar in Japanese modern rice cultivation. Statistics for Japanese rice cultivation in 2014 shows that 36% of paddy fields are planted with Koshihikari and the other popular Japanese cultivars such as Hitomebore, Hinohikari, Akitakomachi and Nanatsuboshi are all derivatives from Koshihikari [71]. It would not be a mere coincidence that Koshihikari, together with IR58, the only ‘IR’ line bred by International Rice Research Institute and that was included in World Rice Core Collection, were both hyper-tolerant to oxidative stress. Improved anti-oxidative capacity may aid growth of rice plants exposed to various stresses in the field. One of the important characteristic of modern cultivars is their eating quality. Then, is there any possibility that anti-oxidative capacity improves eating quality by protecting rice grains from oxidation? For instance, superoxide dismutase and catalase ameliorate postharvest physiological deterioration in cassava, wheat *tasg1* mutant possesses oxidative stress tolerance and show delayed senescence, and paraquat tolerance is positively correlated with longevity of *Drosophila* strains [72–74]. Longevity of rice plant (delayed leaf senescence) will also cause increase in photosynthesis and results in higher rice yield.

Finally, this study was performed in greenhouse and laboratory controlled conditions, thus precautions should be taken into consideration when moving to paddy field. We anticipate that rice breeding aiming to enhance anti-oxidative capacity based on fluorescence analysis will contribute to further improvement of rice cultivars.

## Conclusions

High-throughput estimation of oxidative stress tolerance of rice leaves was performed in this study. This consists of direct treatment of leaf discs with low concentration of methyl viologen. Damage to leaf discs was estimated by chlorophyll fluorescence. Factorization of *F*v/*F*m into *qPI* and *qSlow* was an effective approach for precise comparison between rice cultivars. Furthermore, chlorophyll *a*/*b* ratio was found to be a potential new indicator which can evaluate rice leaf damage under oxidative stress. A large difference in oxidative stress tolerance between rice cultivars was reported. In addition, modern cultivars tended to possess higher tolerance to oxidative stress. These data are useful for breeding of rice cultivars having stronger tolerance to oxidative stress.

## Additional file

**Additional file 1.** Numerical data used to prepare figures. All numerical data used to prepare figures are provided in this file, in the order they appear in figures.

## Abbreviations

*F*v/*F*m: maximum quantum yield of photosystem II
PPFD: photosynthetic photon flux density
*qPI*: a chlorophyll fluorescence parameter estimating the size of photochemistry after treatment relative to the size of photochemistry before treatment
*qSlow*: a chlorophyll fluorescence parameter estimating the size of slow-relaxing non-photochemical quenching relative to the size of basal dissipation
